# Experimental and numerical study of the failure process and energy mechanisms of rock-like materials containing cross un-persistent joints under uniaxial compression

**DOI:** 10.1371/journal.pone.0188646

**Published:** 2017-12-12

**Authors:** Rihong Cao, Ping Cao, Hang Lin, Xiang Fan

**Affiliations:** 1 School of Resources and Safety Engineering, Central South University, Changsha, Hunan, China; 2 School of Civil, Environmental and Mining Engineering, The University of Western Australia, Perth, Australia; 3 School of Highway, Chang’an University, Xi’an, China; Beihang University, CHINA

## Abstract

Joints and fissures in natural rocks have a significant influence on the stability of the rock mass, and it is often necessary to evaluate strength failure and crack evolution behavior. In this paper, based on experimental tests and numerical simulation (PFC2D), the macro-mechanical behavior and energy mechanism of jointed rock-like specimens with cross non-persistent joints under uniaxial loading were investigated. The focus was to study the effect of joint dip angle α and intersection angle γ on the characteristic stress, the coalescence modes and the energy release of jointed rock-like specimens. For specimens with γ = 30° and 45°, the UCS (uniaxial compression strength), CIS (crack initiation stress) and CDiS (critical dilatancy stress) increase as α increases from 0° to 75°. When γ = 60° and 75°, the UCS, CIS and CDiS increase as α increases from 0° to 60° and decrease when α is over 60°. Both the inclination angle α and intersection angle γ have great influence on the failure pattern of pre-cracked specimens. With different α and γ, specimens exhibit 4 kinds of failure patterns. Both the experimental and numerical results show that the energy of a specimen has similar trends with characteristic stress as α increases.

## Introduction

In most rock engineering cases, there are a large number of discontinuities, such as fissures, joints, and weak surfaces. Joints and fissures in natural rocks have a significant influence on the stability of the rock mass; it is often necessary to evaluate strength failure and crack evolution behavior. The failure process of brittle rock or rock-like materials containing one, two, or three fissures has been extensively investigated and discussed. The investigation of crack initiation and propagation in brittle materials began with a specimen that contains a single joint or fissure. Previous work promoted understanding of the crack initiation around fissure tips [1–6]. Almost all of the scholars arrived at the same conclusion: the inclination angle of the crack has a strong influence on the strength and crack pattern, and there are two kinds of cracks that appear around the crack tips (wing cracks and secondary cracks).

In addition to the cracking behavior of specimens with single cracks, substantial experimental efforts have been devoted to the investigation of crack coalescence behavior in natural rocks or brittle materials [3, 7–24]. For both parallel and un-parallel joints, there are three kinds of coalescence patterns of pre-existing cracks: tensile, shear and mixed cracks. Because in most rock engineering cases there are a large number of joints in the natural rock-mass, the failure modes of multi-fissure specimens have also been investigated by scholars. Many kinds of joint geometry parameters have been considered in previous studies, such as joint inclination [22, 25–27], distance [22, 26, 28–30], continuity factor [22, 25, 29–30] and joint overlap [26]. There are many kinds of failure modes appearing in multi-fissure specimens with different kinds of joint geometry: splitting, stepped path, shearing, block rotation and planar.

With the rapid development of computer science, several numerical methods have been suggested to simulate crack initiation and coalescence. These methods include the FEM [31–38], DDA [39–42], and NMM [43–45]. The emergence of the discrete element method is a major advance in the field of simulation of rock mass, especially the software package PFC, which was developed by the ITASCA Consulting Group based on the discrete element method. PFC is widely used to simulate the cracking process of specimens containing cracks [29, 46–50]. The results of models based on the PFC framework show good agreement with experimental results.

Although significant results have been obtained for specimens containing joints under compressive loading conditions, the whole failure process and energy dissipation of specimens with cross non-persistent joints is not yet clear. Apart from parallel or un-parallel joints, in most rock engineering cross joints are one of the most common patterns. Unlike parallel or un-parallel joints, both the inclination and intersection angle in cross joints have a significant influence on the failure behavior of jointed rocks, especially for the rock-mass in pillars or rock slopes. The crossing of joints alters the stress distribution pattern; different kinds of cross joint configuration result in different failure modes compared with the modes observed in previous studies. The investigation of the failure behavior and energy dissipation of cross non-persistent rock fissures will help us better understand the complex mechanical behavior of natural rock-mass. Based on experiments using rock-like material and PFC2D, the mechanical behavior, failure mode and energy dissipation of cross non-persistent rock-like materials is investigated in this paper.

## Experimental testing and numerical model generation

### Specimen preparation and testing

**Fig 1:**
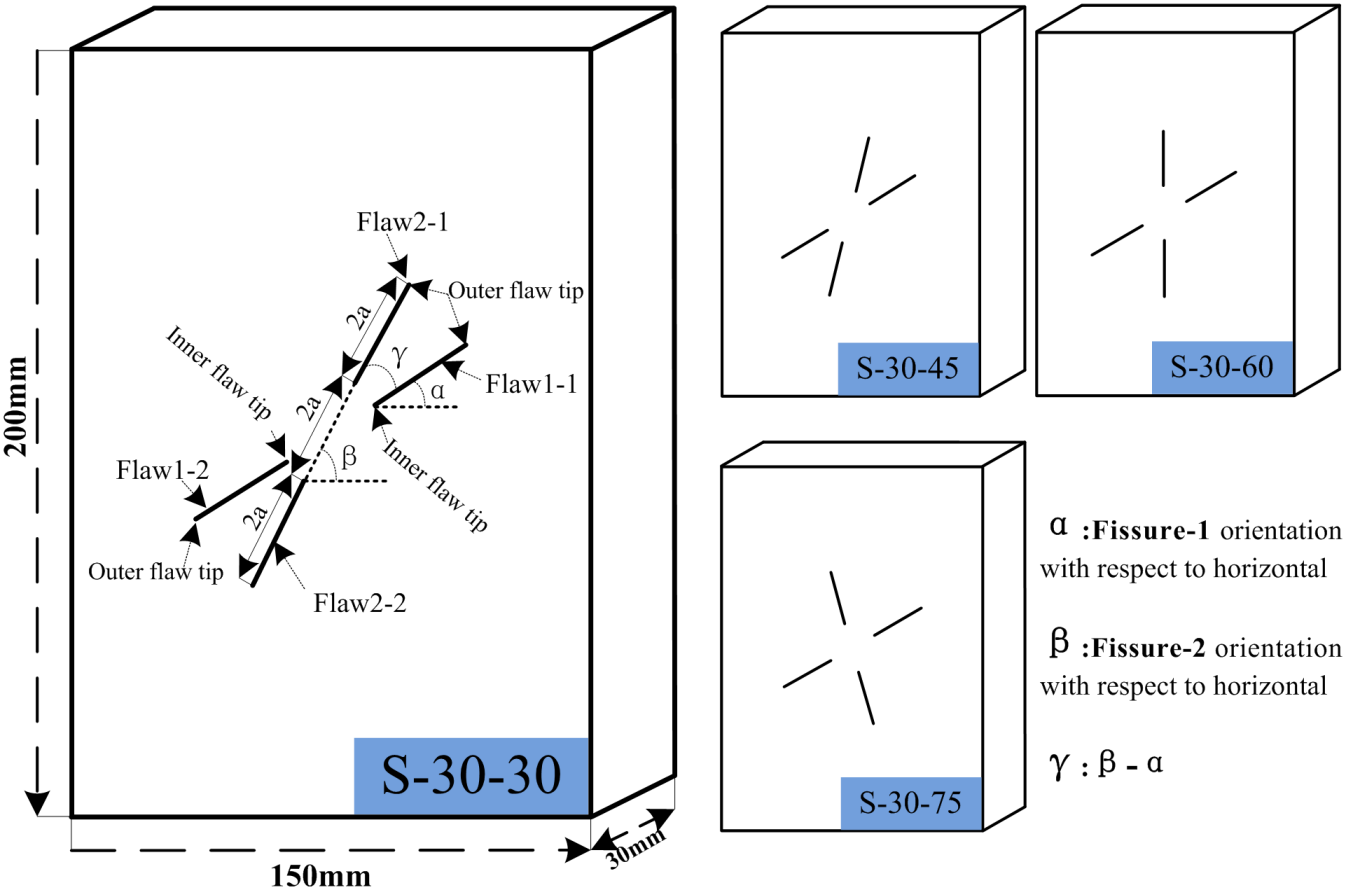
Schematics of fissure geometry configurations in the specimens.

Because sand provides the frictional behavior of the modeling material and because cement is similar to the adhesive materials in natural rocks, white cement and sand mixture has been accepted by scholars for modeling the failure behavior of fractured rocks [9, 10, 25]. In this research, rock-like specimens are made of white cement, water and sand; the volume proportions are V (water): V (white cement): V (silica sand) = 1: 1: 2. As shown in Fig 1, the dimensions of the specimens are 200 (height) × 150 (width) × 30 (thickness) mm. The length of the joint is 30 mm (2a), and it is created by inserting a mica sheet into the fresh cement mortar paste. Before testing, all of the specimens are placed in a standard curing box for 28 days. The strength parameters (average value) for intact materials are as follows: unit weight (γ_m_ = 2.159 g/cm^3^), Young’s modulus (E_m_ = 3.242 GPa), uniaxial compressive strength (UCS = 8.01 MPa), and Poisson’s ratio (*v* = 0.2371).

Joint geometry in this study is defined by two geometrical parameters as shown in Fig 1: Joint set-1 inclination angle α and intersection angle γ (β-α) (Fig 1). The Joint set-1 inclination angle α varies from 0°, 30°, 45°, 60° and 75°. The intersection angle γ varies from 30° to 75° by increments of 15°. Table 1 provides the joint geometry information for all the specimens in this study. Fig 1 shows the joint geometry configurations generated for α = 30 as a set of examples (S-30-30, S-30-45, S-30-60, S-30-75).

**Table 1:** Geometric shape of the four flaws in the specimens.

Number	Specimen ID	α	γ	Number	Specimen ID	α	γ
1	S-0-30	0	30	11	S-45-60	45	60
2	S-0-45	0	45	12	S-45-75	45	75
3	S-0-60	0	60	13	S-60-30	60	30
4	S-0-75	0	75	14	S-60-45	60	45
5	S-30-30	30	30	15	S-60-60	60	60
6	S-30-45	30	45	16	S-60-75	60	75
7	S-30-60	30	60	17	S-75-30	75	30
8	S-30-75	30	75	18	S-75-45	75	45
9	S-45-30	45	30	19	S-75-60	75	60
10	S-45-45	45	45	20	S-75-75	75	75

**Fig 2:**
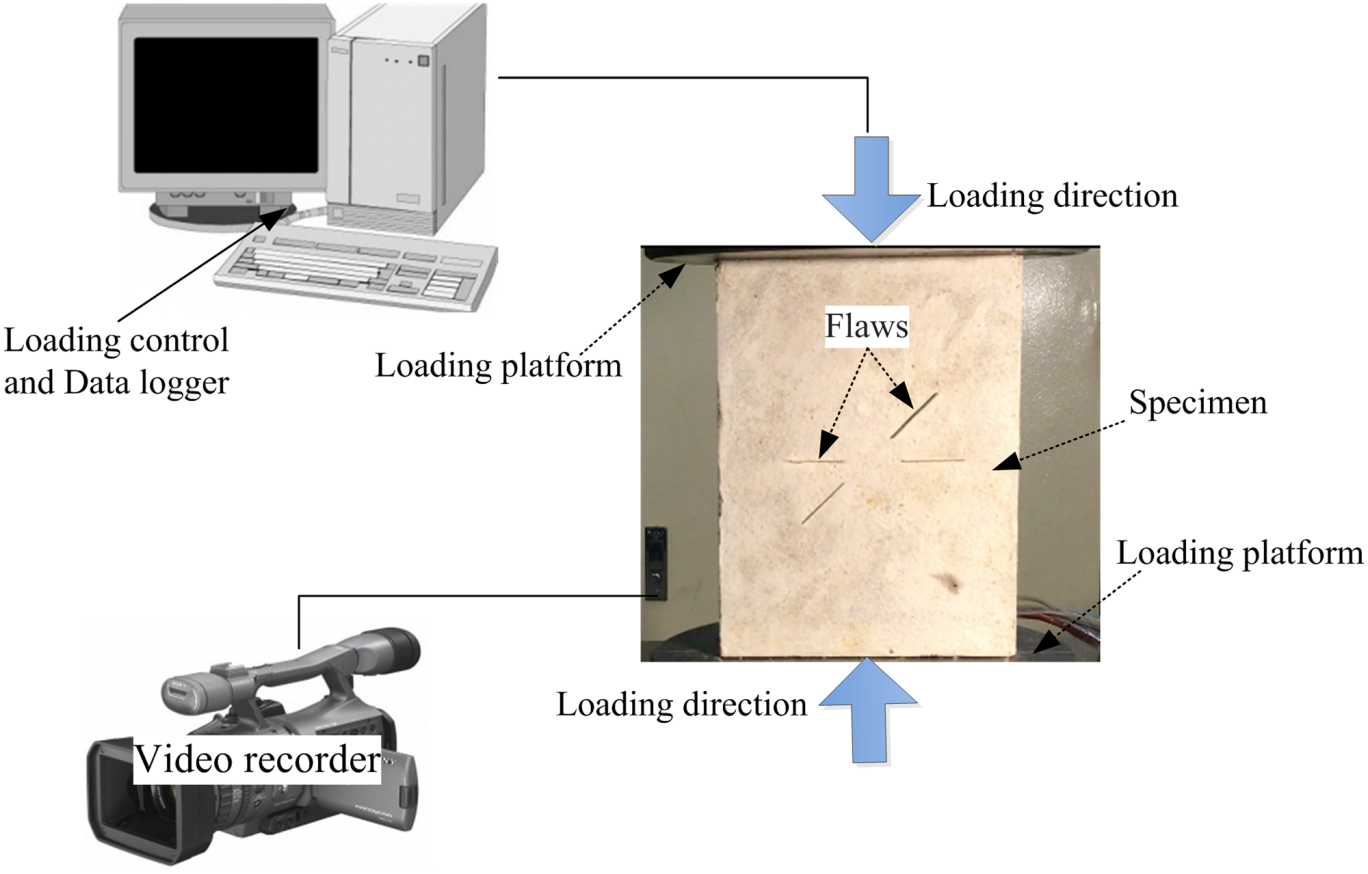
Specimen and the layout of the loading system.

The uniaxial compression tests are conducted using a servo control uniaxial loading instrument together with a DCS-200 control system. During testing, the specimen is sandwiched between the loading platforms (Fig 2). All tests are conducted with displacement-controlled conditions at an average loading rate of 0.2 mm/min; the specimens are loaded under compression until failure.

### Micro-parameter calibration and model generation

In PFC, material is modeled as collections of particles. Each particle contacts its neighboring particles. There are two kinds of contact regimes between particles, namely, a contact-bond model (CPM) and a parallel-bond model (PBM), and both models have their own characteristics. The parallel bond model has been widely accepted by many researchers for modeling rock-like material, because in the parallel bond model, the bond of the PBM is depicted as a rectangle of cementitious material that can transmit both force and moment between particles [50–51]. In the PBM, bond breakage results in immediate decrease in macro stiffness because the stiffness is contributed by both contact stiffness and bond stiffness [50–51].

The parallel bond model describes the constitutive law of the cementitious material deposited between two balls [50–51]. The micro-parameters in PFC2D are determined through “trial and error”. In the previous works, almost all of the micro-parameters are obtained by this method. The micro-parameters in this study are shown in Table 2. A comparison between the experimental and numerical results for macro-parameters of intact material is provided in Table 3. Table 3 shows that the simulated UCS and Young’s modulus of intact rock-like specimens are similar or equal to those obtained experimentally.

**Table 2:** Microscopic parameters for rock mass.

Micro-parameters	Values	Remarks
Minimum mean radius (mm)	0.25	Uniform distribution
*R*_max_/*R*_min_	1.66	
Particle contact modulus, *E*_c_ (GPa)	2.45	
Particle normal/shear stiffness	2.7	
Particle friction coefficient, *μ*	0.5	
Parallel bond modulus, (GPa)	2.45	
Parallel bond normal/shear stiffness	2.7	
Parallel bond normal strengths, mean (MPa)	5.53	Normal distribution
Parallel-bond normal strength, standard deviation (MPa)	0.6	10.84% of mean value
Parallel bond shear strengths, mean (MPa)	5.53	Normal distribution
Parallel-bond shear strength, standard deviation (MPa)	0.6	10.84% of mean value

**Table 3:** Comparison of experimental and numerical results for intact material macro-mechanical parameters.

	Experimental results	Numerical results
Uniaxial compressive strength, UCS (MPa)	8.104	8.136
Young’s modulus, E (GPa)	3.242	3.173
Poisson ratio	0.2371	0.2419

**Fig 3:**
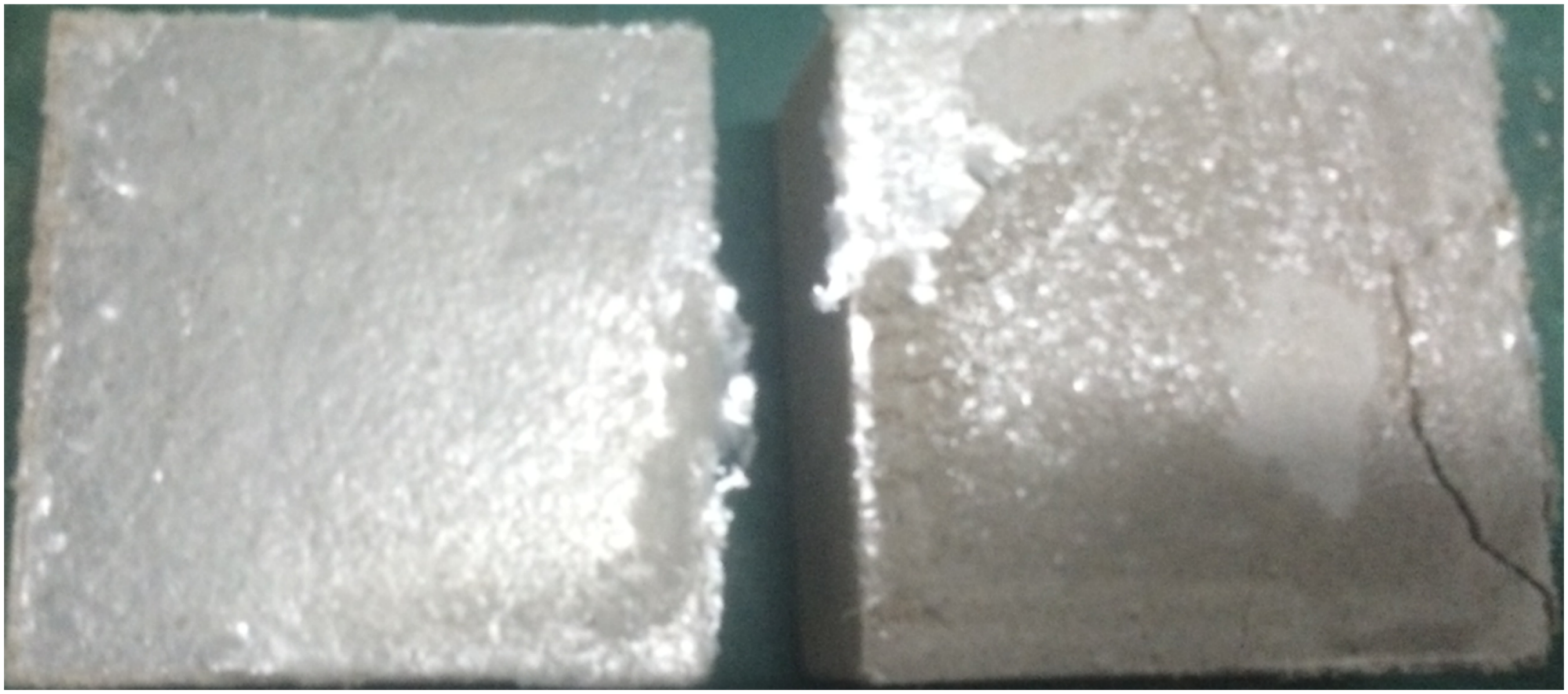
Failure mode of a flat joint with direct shear.

To obtain the mechanical properties of the interface between cemented material and the mica sheet, direct shear tests were conducted. Based on the experimental results, the cohesion (C_j_) and friction angle (φ_j_) of the cemented material and the mica sheet are 18.2 KPa and 11°, respectively. The failure of the cement-mica sheet interface is shown in Fig 3. Usually, the micro-mechanical parameter values assigned for particles that represent joints are smaller than those for the particles that represent intact material. During the curing period, the humidity was controlled at 80%. The mica sheet is composed of multi-layer paper-like material, and after absorbing water, it separates easily during testing. The strength of the mica sheets is similar to that of paper. Therefore, the parallel-bond normal and shear strengths were set as 0.

To determine other micro-parameters of joint particles, direct shear tests under constant normal stress tests were conducted on numerical models; the normal stress values were 0.2 MPa, 0.4 MPa, 1.0 MPa, 1.5 MPa and 2.0 MPa. Like the calibration process for UCS and E, the micro-mechanical parameters for joints are determined by a series of “trial and error” processes (as shown in Table 4).

**Table 4:** Microscopic parameters for joints.

Joint particle friction coefficient	0.08
Joint particle normal stiffness, (N/m)	250
Joint particle shear stiffness, (N/m)	250
Joint particle normal bond strength, (MPa)	0
Joint particle shear bond strength, (MPa)	0

**Fig 4:**
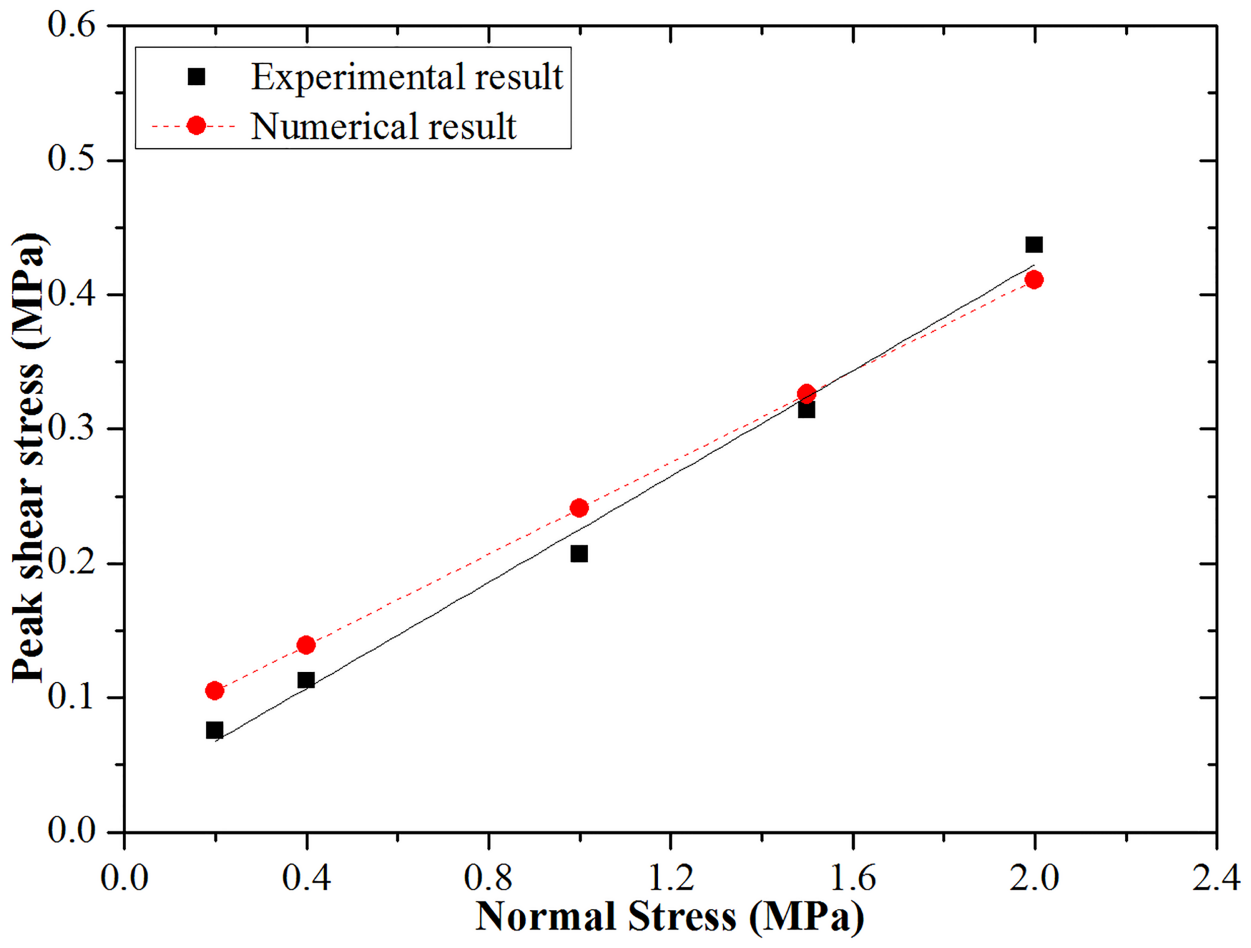
Joint peak shear stress comparisons of experimental and numerical results.

A comparison of the experimental and numerical results for peak shear stress of a flat joint is provided in Fig 4. Fig 4 indicates that the simulated peak shear stress of the flat joint specimens agree with those obtained experimentally.

**Fig 5:**
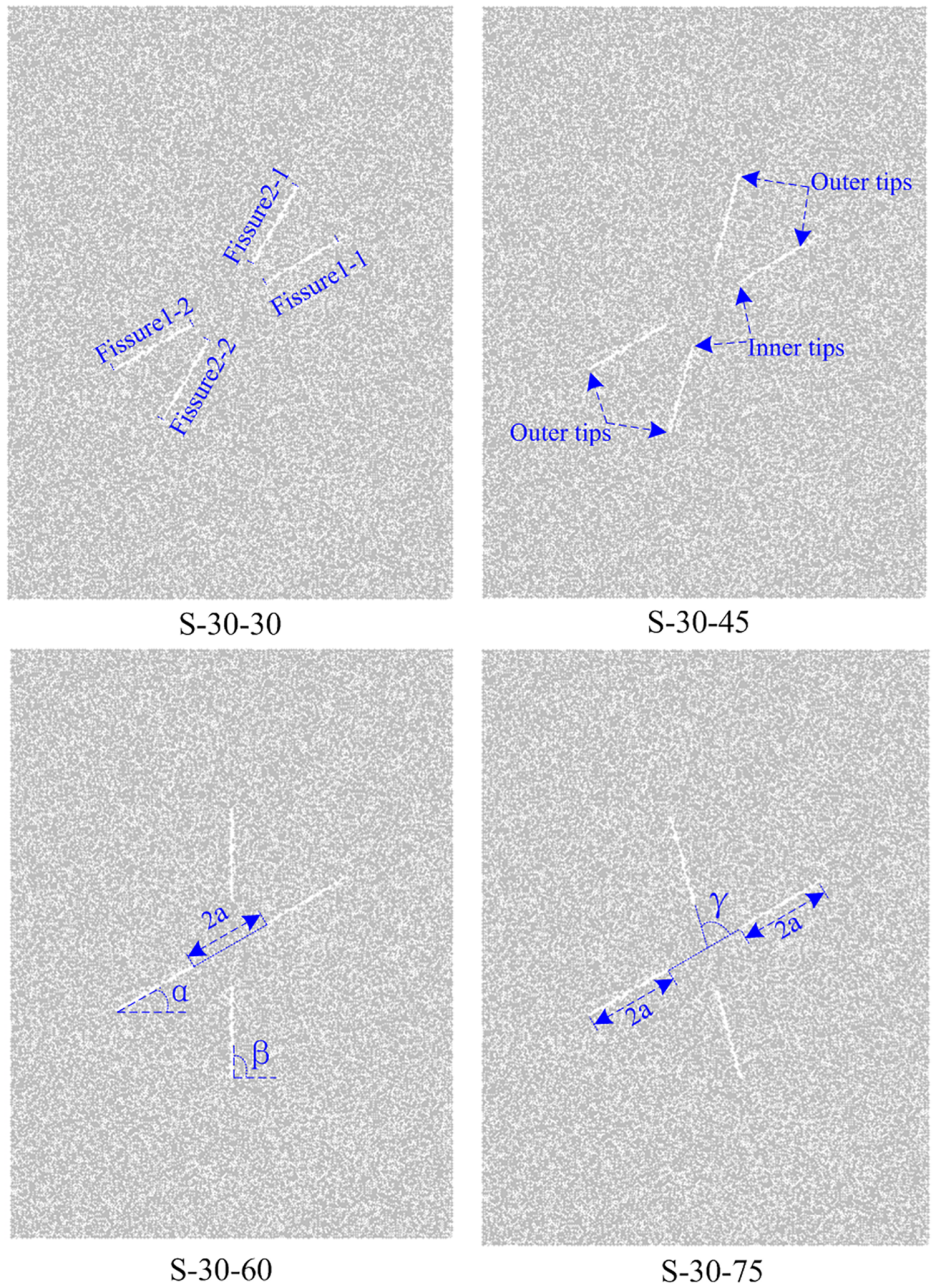
Numerical specimens containing two cross-fissures. α is the angle of fissure 1(α = 30°), β is the angle of fissure 2 (β = 30°, 45°, 60°, and 75°), γ is the angle of fissure 1 and fissure 2 (γ = β-α), and 2a is the fissure length and ligament length (2a = 30 mm).

Fig 5 shows the numerical jointed specimen generated by PFC2D; the scale of the specimen is equal to that of the specimens in the experiment (width and height are 150 mm and 200 mm, respectively). As seen from the specimens, the gray circles are particles of intact material, and the white particles represent joints. A joint in the numerical specimens is generated by assigning a dip angle and dip direction. The length of the joint and ligament are the same as those in the experiments (2a = 30 mm).

## Stress analysis

**Fig 6:**
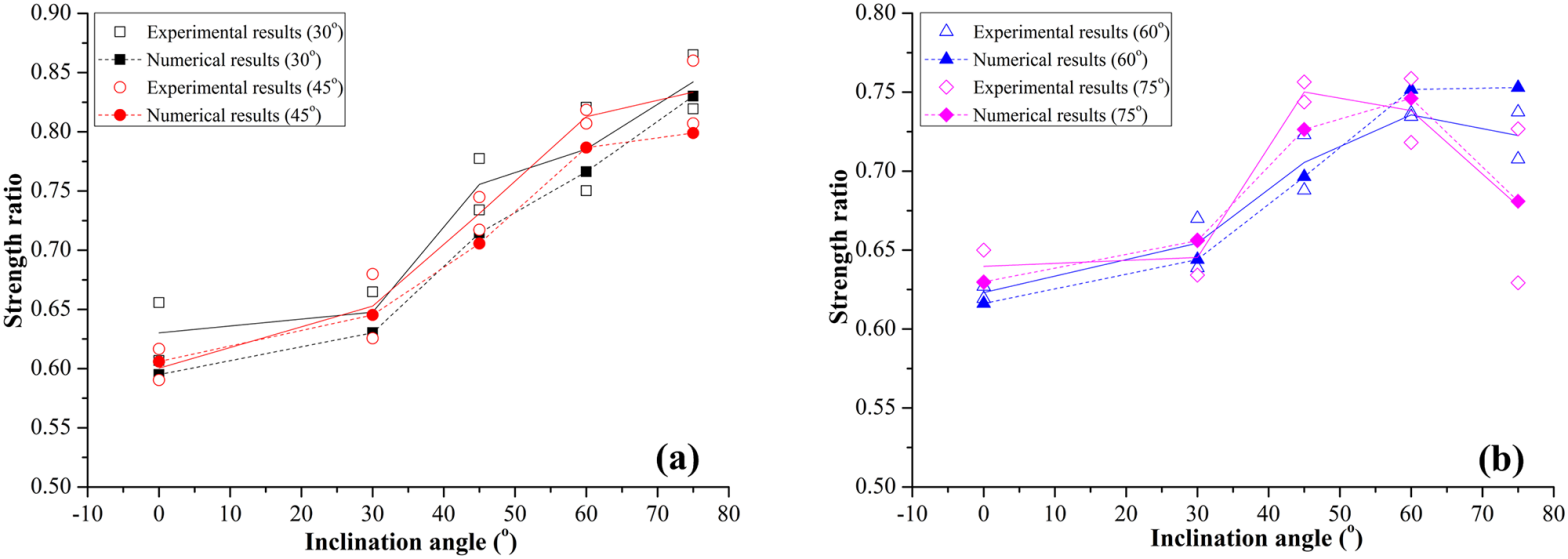
Effect of inclination angle of fissures on *P*r. (a) Experimental value for specimens with γ = 30° and 45°; (b) Experimental value for specimens with γ = 60° and 75°.

Fig 6 shows the characteristic stress in jointed rock-like specimens with different α and γ. The characteristic stress includes UCS, CIS and CDiS. Shown in Fig 6 is the peak strength ratio (*P*r) for experimental and numerical results. The peak strength ratio is the ratio of UCS_J_ and UCS_I;_ UCS_J_ and UCS_I_ are the peak strength values of jointed specimens and intact specimens.

$$\text{Peak}\;\text{strength}\;\text{ratio}\;(\text{Pr})\;=\;\frac{{\text{UCS}}_{\text{J}}}{{\text{UCS}}_{\text{I}}}$$ (1)

In Fig 6, the *P*r numerical results show a similar trend as the experimental results. This trend indicates that the numerical model can simulate the jointed specimen’s mechanical behavior favorably. Fig 6 shows the relationship between *P*r and α for specimens with different intersection angles γ. The joint set-1 inclination angle α has a very high influence on *P*r. For the specimens with γ = 30° and 45°, as α increases, the value of *P*r exhibits an obvious increasing trend (Fig 6(a)). When γ = 60° and 75°, the value of *P*r increases as α increases from 0° to 60° and decreases when α exceeds 60°(Fig 6(b)). The inclination α has a great influence on the mechanical properties of a jointed specimen. For a specimen with lower intersection value, the strength of the specimen is mainly influenced by the joint with higher inclination. The inclination angle of Joint-2 increases with the increase of α. The peak strength ratio shows an increasing trend. For a specimen with higher intersection angle, when the inclination angle of Joint set-1 (α) changes from 0° and 45°, the inclination of Joint-2 also shows an increasing trend. When the α value is 45° and 60°, both Joint set-1 and Joint set-2 are closer to the direction of maximum principal stress; the strength ratio is higher than in other conditions.

The CIS and CDiS are obtained from numerical simulation. The critical dilatancy stress (CDiS) is the stress representing the turning point on the stress-volumetric strain curve; the volumetric strain in PFC is recorded by measurement circle. The CDiS ratio is the ratio of CDiS_J_ and CDiS_I;_ CDiS_J_ and CDiS_I_ are the critical dilatancy stress values of a jointed specimen and intact specimen.

$$\text{CDiS}\;\text{ratio}\;(\text{CDiSr})\;=\frac{{\text{CDiS}}_{\text{J}}}{{\text{CDiS}}_{\text{I}}}$$ (2)

For the CIS, because crack initiation is based on homogeneous rock, it is difficult to locate the crack initiation point on stress-strain curves. In this study, the crack initiation stress (CIS) measured during a uniaxial test on a specimen is defined as the axial stress where there is a specified fraction (1% in the simulations described in Potyondy 2007 [50]) of N_f_; N_f_ is the total number of cracks in the model at the point when the peak strength has been obtained. Like the UCS and CDiS, the CIS ratio is the ratio of CIS_J_ and CIS_I_:

$$\text{CIS}\;\text{ratio}\;\text{(CISr)}\;=\;\frac{{\text{CIS}}_{\text{J}}}{{\text{CIS}}_{\text{I}}}$$ (3)

**Fig 7:**
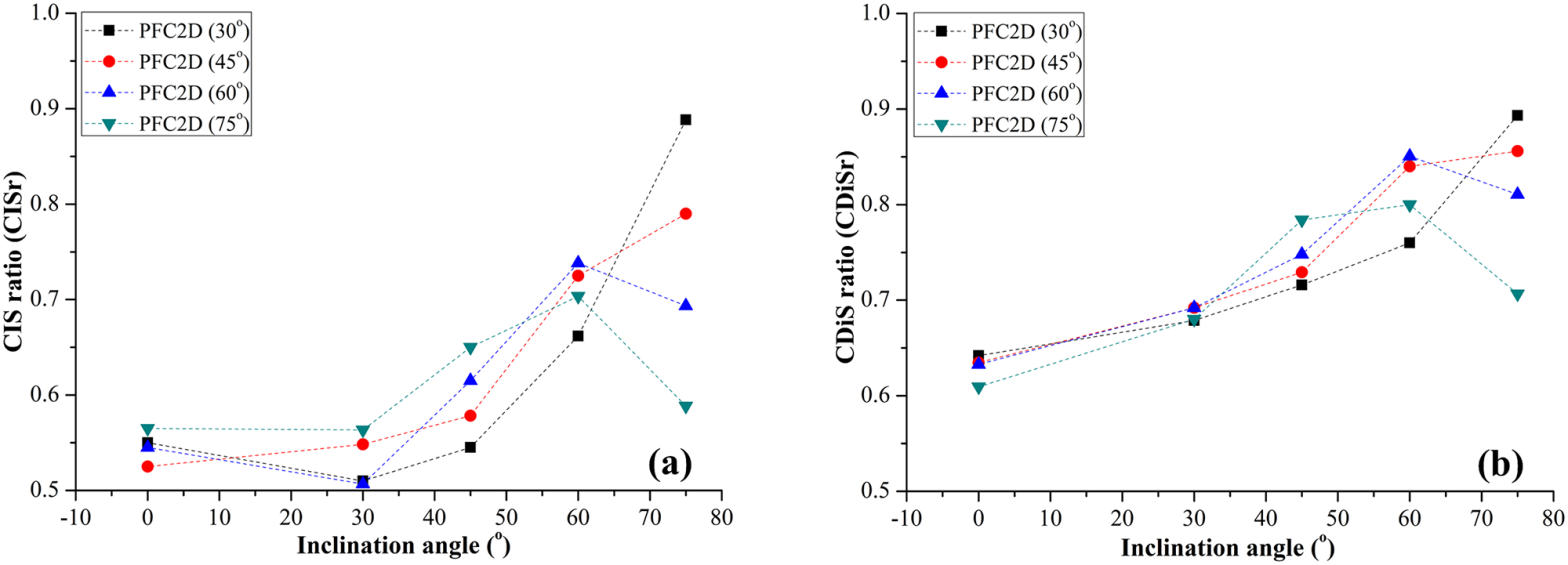
Effect of inclination angle of fissures on characteristic stress. (a) CISr; (b) CDiSr.

In Fig 7(a) and 7(b), for the specimens with γ = 30° and 45°, both CISr and CDiSr have an increasing trend as α increases. The variation of characteristic stresses can be divided into two parts. When α changes from 0° to 30°, the CIS (Fig 7(a)) and CDiS (Fig 7(b)) change slightly. When α changes from 30° to 75°, the characteristic stress grows significantly. For the specimens with intersection angle γ of 60° and 75°, the values of CISr (Fig 7(a)) and CDiSr (Fig 7(b)) increase as α increases from 0° to 60° and decrease after α exceeds 60°. The variation in characteristic stresses can be divided into three parts. The first is from 0° to 30° (fluctuates slightly); the second part is from 30° to 60° (grows significantly); the third part is from 60° to 75° (declines). Clearly, the characteristic stresses (CIS and CDiS) exhibit similar trends as UCS.

## Failure pattern

### Failure pattern based on experimental tests

**Fig 8:**
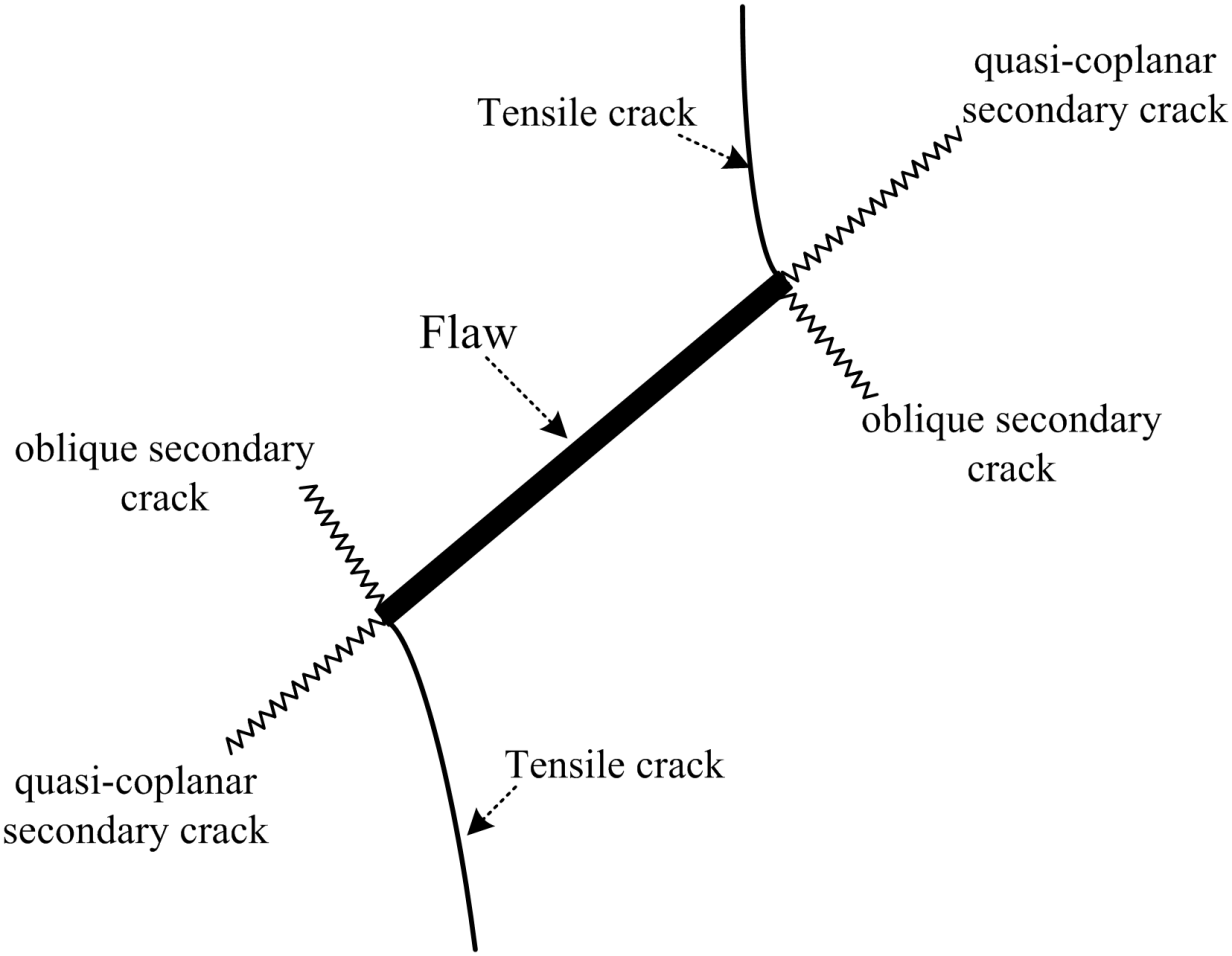
Crack types observed in pre-flawed specimens under compression.

**Fig 9:**
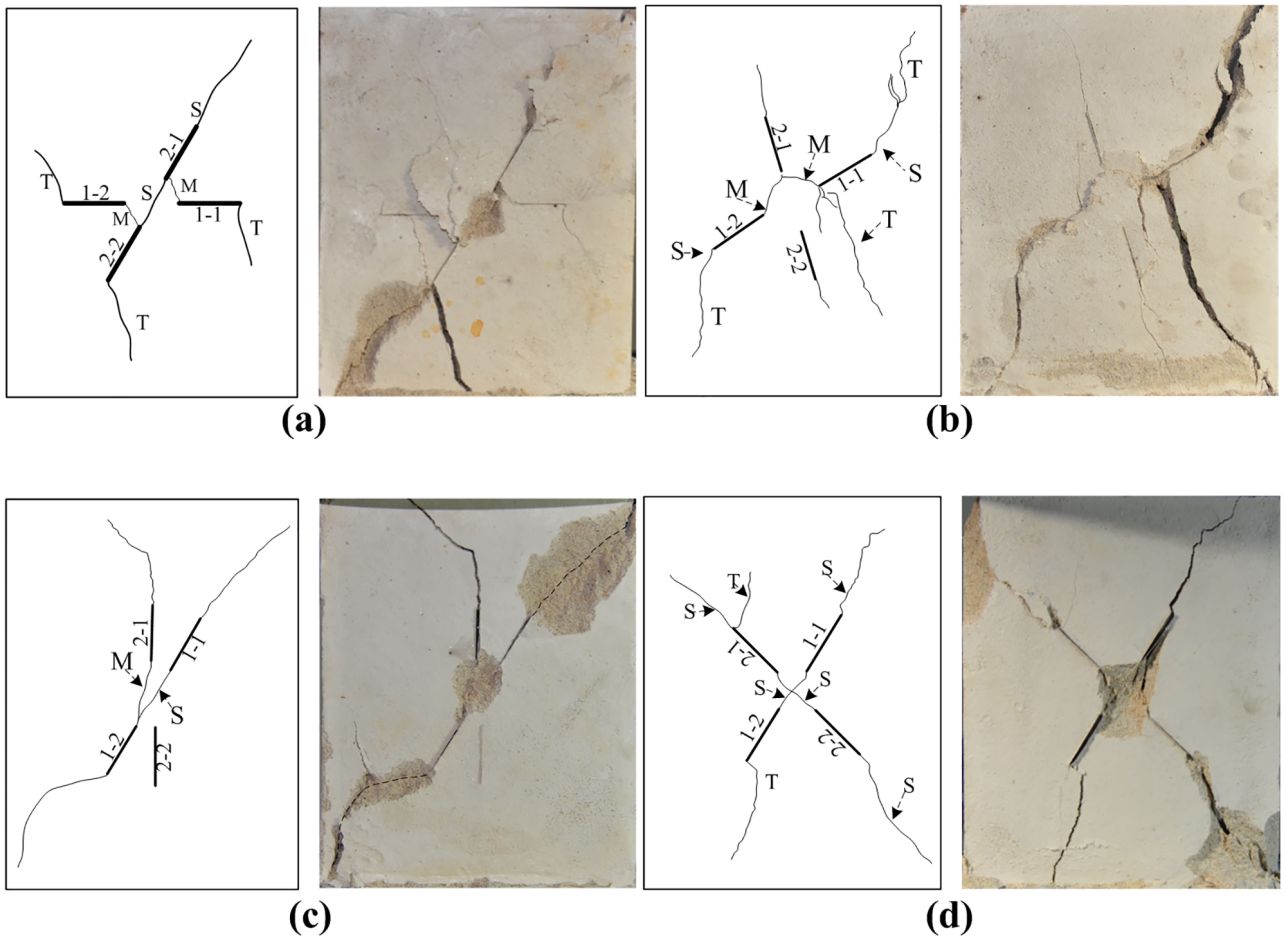
Four different patterns of failure mode observed in our experiments. (a) Pattern I; (b) Pattern II; (c) Pattern III; (d) Pattern IV. The letters S, M and T indicate the shear, mixed shear/tensile, and tensile modes of coalescence, respectively.

As mentioned above, the investigation of crack initiation and propagation in brittle materials began with a specimen that contained a single crack or fissure. Almost all of the scholars arrived at the same conclusion: there are two types of cracks initiated from the tips of joints, namely, wing cracks and secondary cracks as shown in Fig 8 [1, 8, 10, 14, 52–53]. For co-planar joints, when the inclination angle of a joint is smaller than 30°, the joint will not form coalescence, and the failure of the specimen results from the propagation of tensile cracks [5, 17, 21–22, 25, 30, 52]. For a specimen with joint inclination more than 30°, the joint forms coalescence via shear cracks [5, 17, 21–22, 25, 30, 52]. However, when a joint is parallel to the loading direction, the failure of the specimen is very similar to an intact specimen. The joints in the specimen have difficulty forming coalescence [5, 17, 21–22, 25, 30, 52]. For cross non-persistent joints, there are two sets of planar joints in a specimen. Under loading, the specimens will have different kinds of failure modes. Comparing with previous works, our study shows that distinctly different coalescence occurs between cross joints. It is clear that both the inclinational angle α and intersection angle γ have great influence on the failure pattern of pre-cracked specimens. Fig 9 shows the crack initiation and coalescence behavior of rock-like samples containing cross non-persistent joints under uniaxial loading. With different α and γ, the specimens exhibit four failure patterns.

#### Pattern-I

As shown in Fig 9(a), there is no coalescence between joint 1–1 and 1–2, and the coalescence pattern between 2–1 and 2–2 is co-planar shear crack. The experimental results show that there are mixed cracks appearing between joint sets 1 and 2. This kind of failure pattern mainly occurs in specimens with α = 0°. Because the joint set-1 is perpendicular to the loading direction, under loading, new cracks developed from the tips of pre-existing joints and propagate along the direction of maximum principle stress. No coalescence occurs between joint set-1. The intersection angle γ varies from 30° to 75^o^; joint set-2 easily forms coalescence through the shear crack. Because the rock bridge between joint sets 1 and 2 is short, joint sets 1 and 2 will form coalescence at the later stage of loading.

#### Pattern-II

In this Pattern, there is no coalescence between joint sets 1 and 2. Joint 1–1 and 1–2 connect with joint 2–1 through mixed cracks (Fig 9(b)). Under loading, shear cracks develop from tips of joint 1–1 and 1–2. As loading continues, shear cracks gradually turn to tensile cracks. During the residual stage, the tensile cracks reach the edge of the specimen and result in the overall failure of the specimens. In this pattern, the pre-existing joints do not form coalescence via shear crack. The reason for this phenomenon is the rock bridge between 1–1 and 2–1 is shorter than between 1–1 and 1–2. Under loading, the rock bridge between 1–1 and 2–1 more easily forms coalescence. Joint set-2 is almost parallel to the direction of maximum principle stress; therefore, it is difficult to form coalescence through shear or tensile cracks. Under uniaxial compression, the pre-existing joints between the two sets tend to link with each other via mixed cracks.

#### Pattern-III

In this category, the crack coalescence mode in joint set-1 is co-planar shear crack (Fig 9(c)). However, there is no coalescence in joint set-2. Joint 2–1 and 1–2 form coalescence through tensile cracks. There is obvious surface spalling around the cross center of joint sets 1 and 2. Because there is a joint set (set-2) parallel to the loading direction, this joint set has difficulty forming coalescence. Joint set-1 becomes the dominant joint set. Joint set-2 has a little influence on the failure pattern of the specimen; the failure mode of the specimen is mainly controlled by joint set-1. When the inclination of joint set-1 (α) is 60° and 75°, the joints in set-1 easily link with each other through shear cracks. This pattern mainly occurs in specimens with α = 60° and 75°.

#### Pattern-IV

For this pattern, the specimen mainly fails from the propagation of shear cracks. As seen from Fig 9(d), the crack coalescence mode in joint set-1 (1–1 and 1–2) and joint set-2 (2–1 and 2–2) are co-planar shear cracks. There is obvious surface spalling around the cross center of joint sets 1 and 2. The failure plane in this pattern is very similar to pattern III; the coalescence between pre-existing joints are shear mode. Compared to pattern III, the main characteristic of this pattern is the cross-shear failure plane. Because the intersection angle of joint set-1 and set-2 in the horizontal direction is 45° to 75°, each joint set forms coalescence via shear cracks; there are two shear failure planes in the specimen.

Table 5 summarizes the failure patterns of jointed specimens with different α and γ. Table 5 shows that both α and γ have a great influence on the failure pattern of the specimen. Because there is a joint set perpendicular to the loading direction, Pattern I is more prominent in the specimen with α = 0°. Specimens S-30-30, S-30-45 and S-45-30 also belong to Pattern I. Pattern II is more prominent in the specimen with α = 30° and 45°. Apart from specimens S-30-30, S-45-30 and S-45-75, the rest of the specimens belong to this pattern. For the specimens with α = 60°, apart from the S-60-75, all of the specimens belong to Pattern III. When α = 75°, most of the specimens fail from propagation of shear cracks (Pattern IV).

**Table 5:** Initiated crack types of rock-like specimens containing cross-flaws with different α and γ.

	α	0	30	45	60	75
γ						
30		I	I	I	III	III
45		I	I	II	III	IV
60		I	II	II	III	IV
75		I	II	IV	IV	IV

### Typical failure pattern in PFC2D

**Fig 10:**
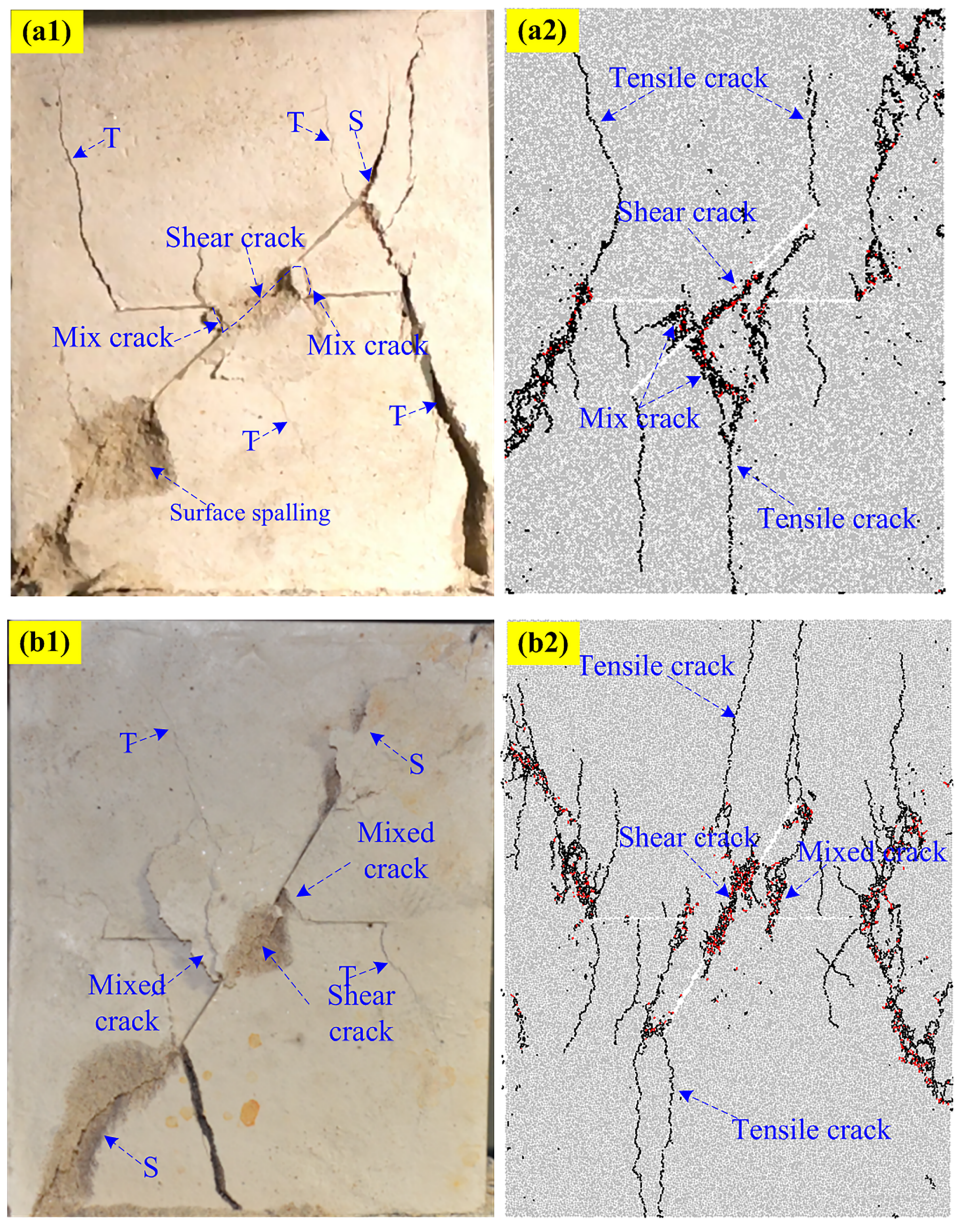
Pattern I comparisons between experimental and numerical results. (a1) Experimental (S-0-30); (a2) Numerical (S-0-30); (b1) Experimental (S-0-60); (b2) Numerical (S-0-60).

**Fig 11:**
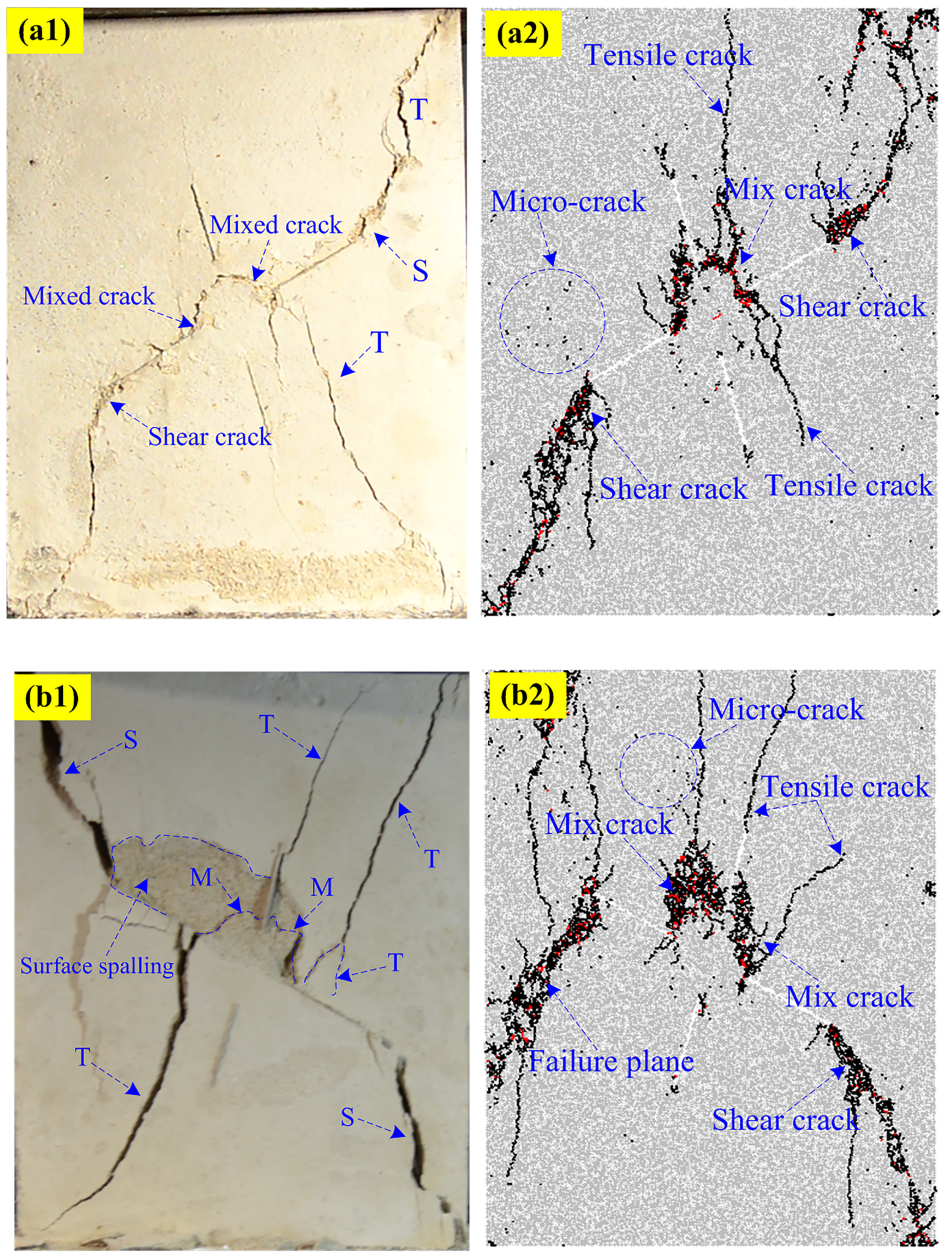
Pattern II comparisons between experimental and numerical results. (a1) Experimental (S-30-75); (a2) Numerical (S-30-75); (b1) Experimental (S-75-75); (b2) Numerical (S-75-75).

**Fig 12:**
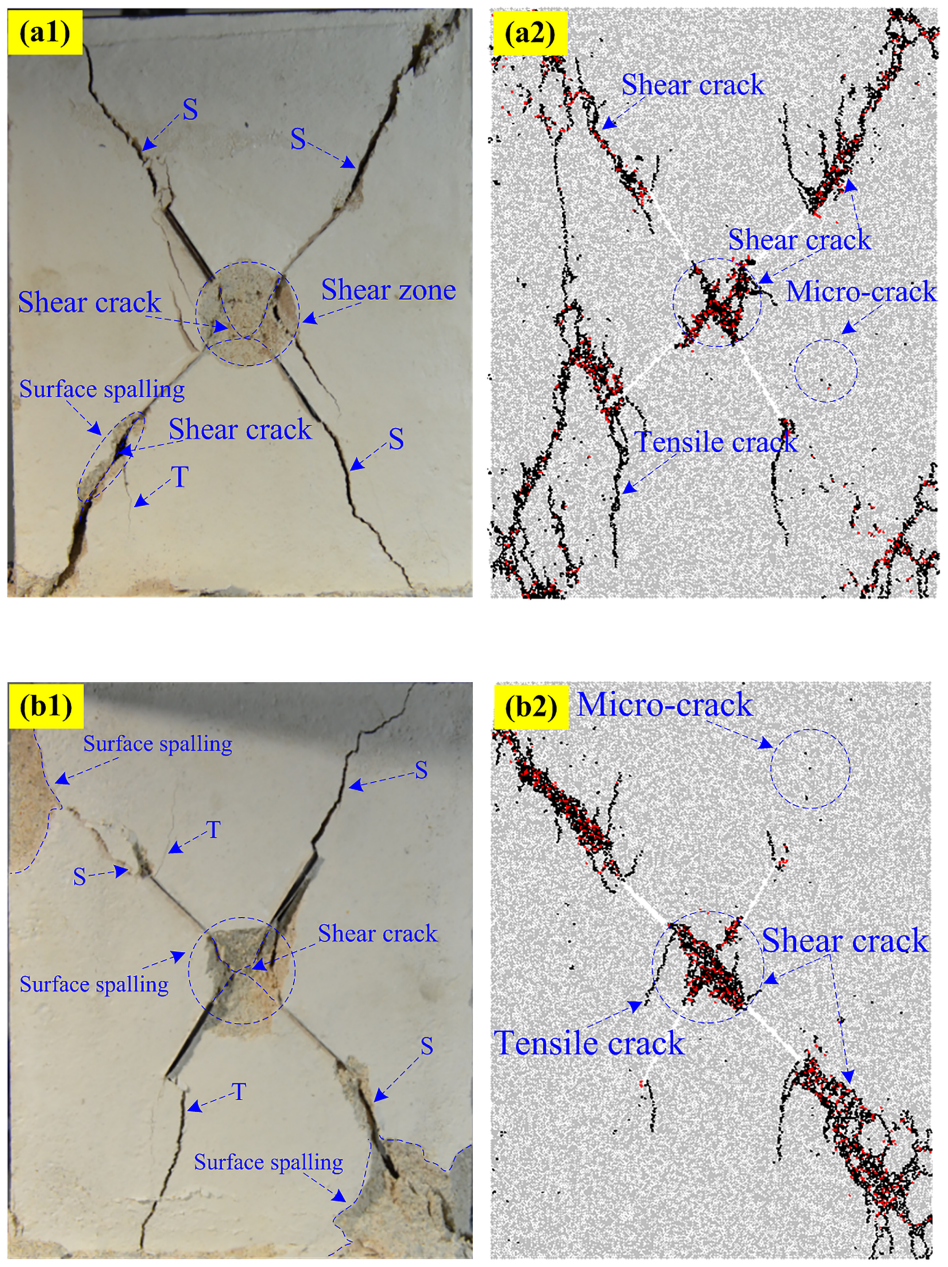
Pattern III comparisons between experimental and numerical results. (a1) Experimental (S-45-75); (a2) Numerical (S-45-75); (b1) Experimental (S-60-75); (b2) Numerical (S-60-75).

**Fig 13:**
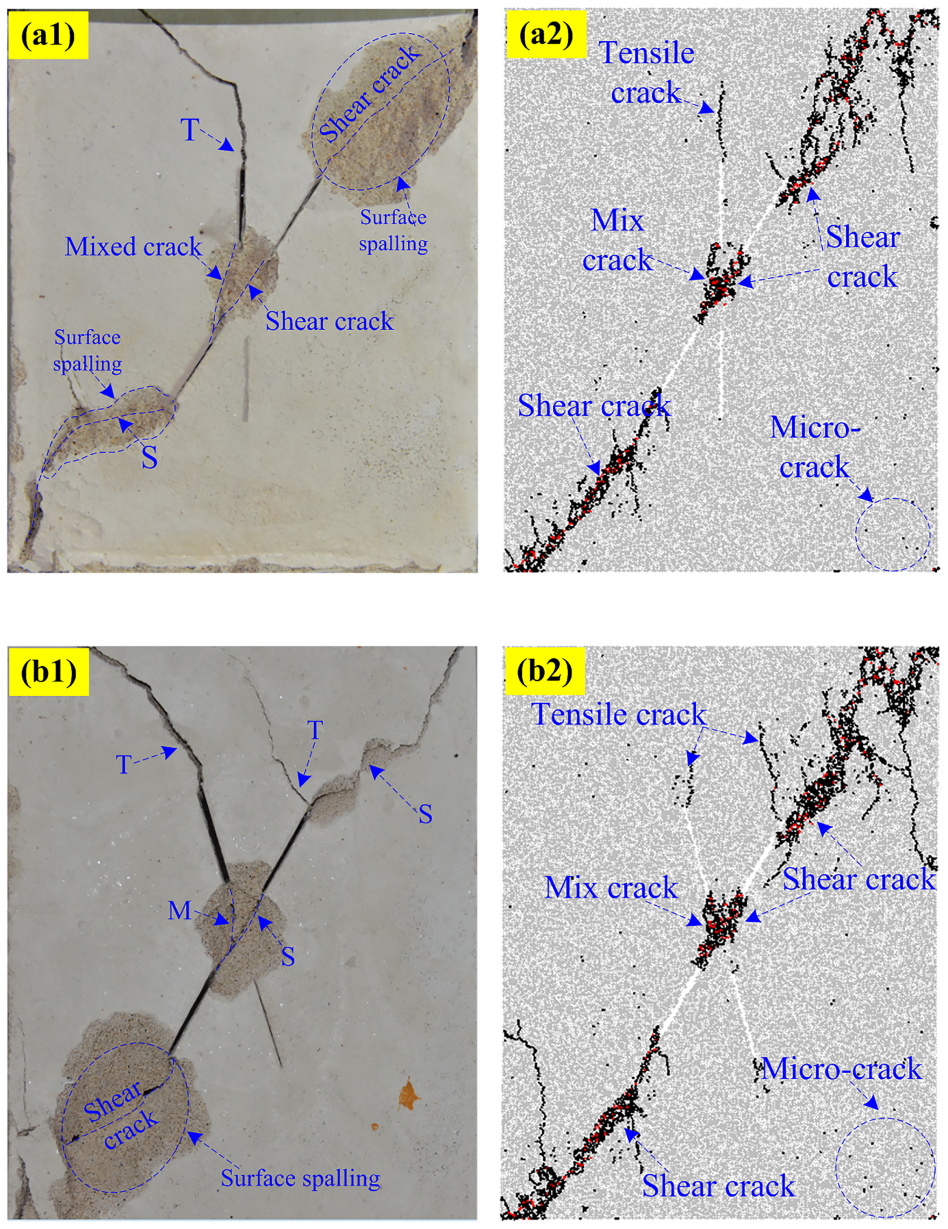
Pattern IV comparisons between experimental and numerical results. (a1) Experimental (S-60-30); (a2) Numerical (S-60-30); (b1) Experimental (S-60-45); (b2) Numerical (S-60-45).

Figs 10–13 shows the five typical failure modes obtained by PFC2D and the corresponding failure modes obtained for the same specimens through rock-like material experiments. There is strong agreement among the four failure patterns obtained in numerical modeling and the experimental results. In the comparison of the pattern I (Fig 10), one set of non-persistent joints links through co-planar shear cracks; the crack coalescence mode between joint sets 1 and 2 belong to mixed cracks. For pattern II (Fig 11), the crack coalescence mode between joint sets 1 and 2 is mixed cracks, and the overall failure of the specimen results from the propagation of shear cracks. Comparison of typical pattern III examples (Fig 12) shows the failure pattern mainly results from shear crack coalescence. It can also be seen from the numerical and experimental results that shear cracks developed from the tips of joints reach the edge of the specimens and result in the overall failure of the specimens. Fig 13 shows the comparison of the numerical and experimental results for pattern IV. Although some of the parts fell outside the specimen, the crack path in the experimental result is very clear and agrees well with the numerical specimens.

## Energy characteristics

The failure of rock or rock-like materials can be viewed as the result of energy conversion. For the uniaxial compression test, before the axial stress reaches peak strength, the absorbed energy is mainly stored as strain energy. Some of energy is consumed by micro-crack generation, propagation and friction of micro defects. Based on the first law of thermodynamics, for a closed system, if a unit volume of material deforms by action of an external force, then the energy can be defined as follows:

$$U=U_{d}+U_{e}$$ (4)

where U_d_ is the unit dissipation energy, and U_e_ is the unit strain energy.

**Fig 14:**
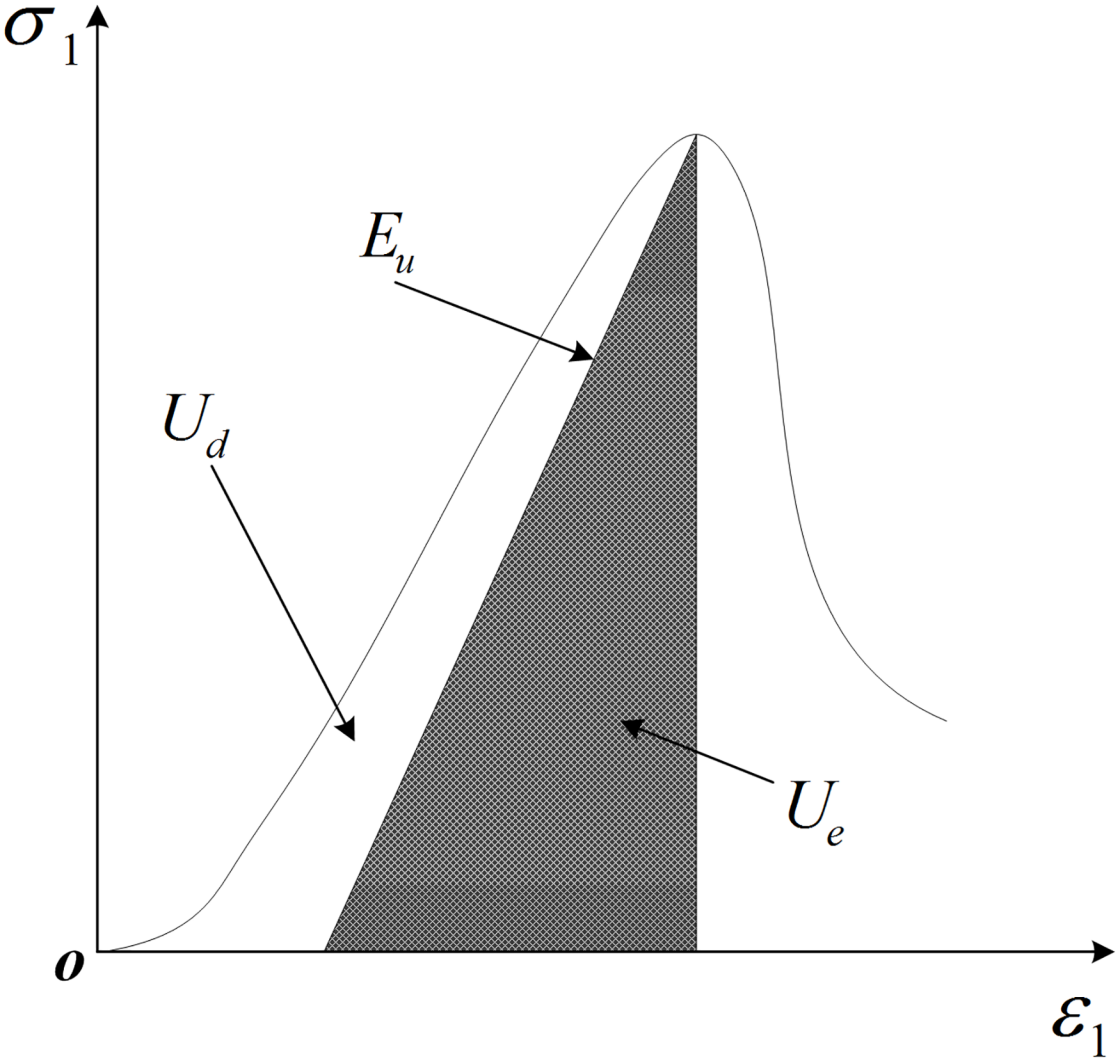
Relationship between dissipation strain energy *U*_d_ and release elastic strain energy *U*_e_ in stress-strain curves.

Fig 14 shows the relationship between dissipation strain energy *U*_d_ and release elastic strain energy *U*_e_ in stress-strain curves. Based on the previous results [54], under compression, the release elastic strain energy *U*_e_ can be calculated as follows:

$$U_{e}=\frac{1}{2E_{u}}\sigma_{1}^{2}$$ (5)

where *E*_u_ is the unloading elastic modulus. In the current research, for calculation convenience, the *E*u is replaced by initial elastic modulus *E*_o_; it is also verified by Liang [55].

For the PFC, the specimen was sandwiched between two walls, and the displacement loading was performed through the top and bottom walls. Because there was no angular displacement in the numerical simulation (PFC2D), the input energy for uniaxial compression in PFC2D can be calculated as follows [56]:

$$E=E_{pre}+(F_{1}\Delta U_{1}+F_{2}\Delta U_{2})$$ (6)

where *E*_pre_ is the input energy in the last calculation step, and *F*_1_ and *F*_2_ are the unbalanced forces on the top and bottom walls, respectively. ΔU_1_ and ΔU_2_ are the displacement increments for the top and bottom walls, respectively. The strain energy (*E*_e_) is stored in the contacts and can be calculated as follows:

$$E_{e}=\frac{1}{2}\sum_{N}\left({\left|F_{i}^{n}\right|}^{2}/k^{n}+{\left|F_{i}^{s}\right|}^{2}/k^{s}\right)$$ (7)

N is the total number of contacts in the mode, and |*F*_*i*_^n^| and |*F*_*i*_^s^| are the normal and shear contact forces, respectively. *k*_n_ and *k*_s_ are the normal and shear-contact shiftiness, respectively.

**Fig 15:**
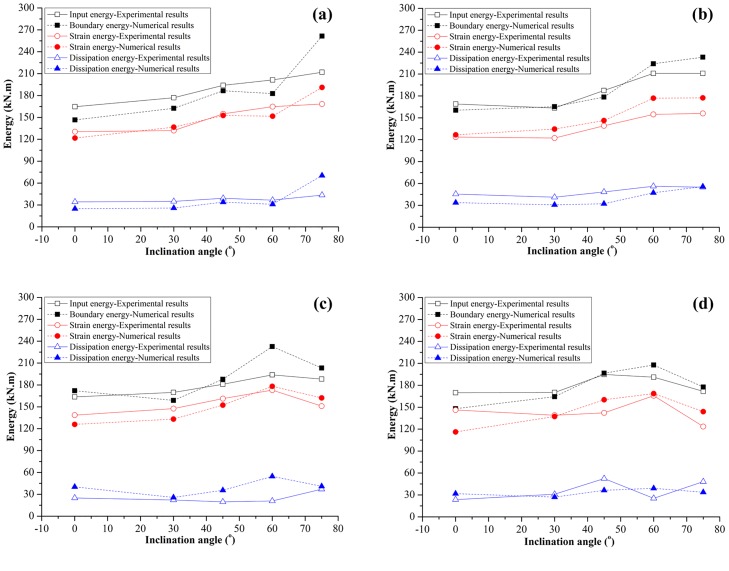
Energy of jointed specimens in laboratory tests and PFC simulation (unit thickness). (a) γ = 30°; (b) γ = 45^o^; (c) γ = 60°; (d) γ = 75°.

Fig 14 shows that the input energy is the area under the axial stress-strain curve. The strain energy is the shaded area under the stress-strain curve. The dissipation energy is the difference between the two areas. In PFC2D, the strain energy and boundary energy can also be recorded by measurement circles; the dissipation energy is the difference between them. All types of energy densities for the set of experimental tests are shown in Fig 15. It can be seen from Fig 15 that the input and strain energy densities exhibit a similar trend with the characteristic stress in Fig 6. The strain energy is significantly greater than the dissipation energy, and the dissipation exhibits some fluctuation with the increases of α. For the numerical simulation, although there is also some difference between experimental and numerical results, all energies in the numerical simulation results show a similar trend with those in the experimental results. As α changes from 0° to 75°, the input energy and strain energy show a similar trend with the UCS of the specimens. The dissipation energy is the lowest of the three and exhibits a little fluctuation with the increase of α. The energies in experimental and numerical results are based on the unit thickness.

## Conclusion

In this paper, the characteristic stress, failure pattern and energy mechanism of jointed rock-like specimens under uniaxial loadings are investigated by laboratory tests and numerical simulation. The following conclusions are drawn from the study.

1. Both joint inclination angle α and intersection angle γ have an influence on stress characteristics stress of a specimen. When the intersection angle γ = 30° and 45°, the UCS, CIS and CDiS increase as α increases from 0° to 75°. However, for γ = 60° and 75°, the UCS, CIS and CDiS increase as α increases from 0° to 60° and decrease after α exceeds 60°.
2. The failure patterns of joint rock-like specimens can be classified into four categories. Both α and γ have strong influence on the failure mode. Pattern I is more prominent in specimens with α = 0°. Pattern II is more prominent in specimens with α = 30° and 45°. For specimens with α = 60°, apart from the S-60-75, all of the specimens belong to pattern III. When α = 75°, most of the specimens fail from propagation of shear cracks (pattern IV).
3. Based on the results of the laboratory tests and PFC2D simulation, the energy mechanism of jointed specimens under uniaxial compression has been analyzed. With increasing α, the input energy and strain energy show a similar trend with the UCS of the specimens. The dissipation energy is the lowest of three and exhibits some fluctuation with the increases of α.

## References

[1] Shen B. The mechanism of fracture coalescence in compression experimental study and numerical simulation. Engineering Fracture Mechanics. 1995; 51(1): 73–85.

[2] Wong RHC, Leung WL, Wang SW. Shear strength study on rock-like models containing arrayed open joints In: Elsworth D, Tinucci JP, Heasley KA (eds) Rock mechanics in the national interest. Swets & Zeitlinger Lisse, The Netherland, 2001; pp 843–849.

[3] Dyskin AV, Sahouryeh E, Jewell RJ. Influence of shape and locations of initial 3-D cracks on their growth in uniaxial compression. Engineering Fracture Mechanics. 2003; 70(15): 2115–2136.

[4] Park CH, Bobet A. Crack coalescence in specimens with open and closed flaws: a comparison. Int J Rock Mech Min Sci. 2009; 46(5): 819–829.

[5] Wong LNY, Li HQ. Numerical study on coalescence of two pre-existing coplanar flaws in rock. Int. J. Solids Struct. 2013; 50, (22–23): 3685–3706.

[6] Camones Mejía LA, Vargas J, Amaral E, Figueiredo D, Peluci R. Application of the discrete element method for modeling of rock crack propagation and coalescence in the step-path failure mechanism. Eng. Geol. 2013; 153: 80–94.

[7] Hoek E, Bieniawski ZT. Brittle fracture propagation in rock under compression. International Journal of Fracture. 1965; 1(3): 137–155.

[8] Li YP, Chen LZ, Wang YH. Experimental research on pre-cracked marble under compression. International Journal of Solids and Structures. 2005; 42(9/10): 2505–2516.

[9] Cao P, Liu TY, Pu CZ, Lin H. Crack propagation and coalescence of brittle rock-like specimens with pre-existing cracks in compression. Engineering Geology. 2015; 187(17): 113–121.

[10] Cao RH, Cao P, Lin H, Pu CZ, Ou K. Mechanical behavior of brittle rock-like specimens with pre-existing fissures under uniaxial loading: experimental studies and particle mechanics approach. Rock Mechanics and Rock Engineering. 2016; 49(3): 763–783.

[11] Yang SQ, Dai YH, Han LJ. Experimental study on mechanical behavior of brittle marble samples containing different flaws under uniaxial compression. Eng. Fract. Mech. 2009; 76: 1833–1845.

[12] Yang SQ, Jing HW. Strength failure and crack coalescence behavior of brittle sandstone samples containing a single fissure under uniaxial compression. Int. J. Fract. 2011; 168: 227–250.

[13] Yang SQ, Yang DS, Jing HW, Li YH, Wang SY. An experimental study of the fracture coalescence behaviour of brittle sandstone specimens containing three fissures. Rock Mech Rock Eng. 2012; 45(4): 563–582.

[14] Yang SQ, Liu XR, Jing HW. Experimental investigation on fracture coalescence behavior of red sandstone containing two unparallel fissures under uniaxial compression. International Journal of Rock Mechanics & Mining Sciences. 2013; 63: 82–92.

[15] Sagong M, Bobet A. Coalescence of multiple flaws in a rock-model material in uniaxial compression. International Journal of Rock Mechanics and Mining Sciences. 2002; 39(2): 229–241.

[16] Tang CA, Lin P, Wong RHC, Chau KT. Analysis of crack coalescence in rock-like materials containing three flaws—Part II: numerical approach. Int J Rock Mech Min Sci. 2001; 38(7): 925–939.

[17] Wong LNY. Crack Coalescence in Molded Gypsum and Carrara Marble (Ph.D.) Massachusetts Institute of Technology. 2008.

[18] Wong RHC, Chau KT. Crack coalescence in a rock-like material containing two cracks. International Journal of Rock Mechanics and Mining Sciences. 1998; 35(2):147–164.

[19] Bobet A, Einstein HH. Fracture coalescence in rock-type materials under uniaxial and biaxial compression. International Journal of Rock Mechanics and Mining Sciences. 1998(a); 35(7):863–888.

[20] Bobet A, Einstein HH. Numerical modeling of fracture coalescence in a model rock material. International Journal of Fracture. 1998(b) 92(3): 221–252.

[21] Chen X, Liao ZH, Peng X. Deformability characteristics of jointed rock masses under uniaxial compression. Int. J. Min. Sci. Technol. 2012; 22: 213–221.

[22] Li HQ, Wong LNY. Influence of flaw inclination angle and loading condition on crack initiation and propagation. Int. J. Solids Struct. 2012; 49: 2482–2499.

[23] Li HQ, Wong LNY. Numerical study on coalescence of pre-existing flaw pairs in rock-like material. Rock Mech. Rock. Eng. 2014; 47: 2087–2105.

[24] Manouchehrian A, Marji MF. Numerical analysis of confinement effect on crack propagation mechanism from a flaw in a pre-cracked rock under compression. Acta Mech. Sinica. 2012; 28(5):1389–1397.

[25] Cao RH, Cao P, Fan X, Xiong XG, Lin H. An Experimental and Numerical Study on Mechanical Behavior of Ubiquitous-Joint Brittle Rock-Like Specimens Under Uniaxial Compression Rock Mech Rock Eng. 2016; 49:4319–4338.

[26] Yang XX, Jing HW, Tang CA, Yang SQ. Effect of parallel joint interaction on mechanical behavior of jointed rock mass models. International Journal of Rock Mechanics & Mining Sciences. 2017; 92: 40–53.

[27] Lin H, Cao P, Wang YX. Numerical simulation of the layered rock mass under triaxial compression test. International Journal of Rock Mechanics and Mining Sciences. 2013; 60, 12–18.

[28] Prudencio M, Jan VSM. Strength and failure modes of rock mass models withnon-persistent joints. International Journal of Rock Mechanics & Mining Sciences. 2007; 44: 890–902.

[29] Bahaaddini M, Sharrock G, Hebblewhite BK. Numerical investigation of the effect of joint geometrical parameters on the mechanical properties of a non-persistent jointed rock mass under uniaxial compression. Computers and Geotechnics. 2013; 49: 206–225.

[30] Fan X, Kulatilake PHSW, Chen X. Mechanical behavior of rock-like jointed blocks with multi-non-persistent joints under uniaxial loading: A particle mechanics approach. Engineering Geology. 2015; 190(14): 17–32.

[31] Lin H, Xiong W, Yan QX. Three-dimensional effect of tensile strength in the standard Brazilian test considering contact length. Geotechnical Testing Journal. 2016; 39: 137–143.

[32] Tang CA, Kou SQ. Crack propagation and coalescence in brittle materials under compression. Eng Fract Mech. 1998; 61:311–24.

[33] Moes N, Dolbow J, Belyschko T. A finite element method for crack growth without remeshing. Int J Numer Meth Eng. 1999; 46:131–50.

[34] Moes N, Belytschko T. Extended finite element method for cohesive crack growth. Eng Fract Mech. 2002; 69:813–33.

[35] Areias P, Rabczuk T. Finite strain fracture of plates and shells with configurational forces and edge rotations. Int J Numer Meth Eng. 2013; 94:1099–1122.

[36] Areias P, Dias-da-Costa D, Alfaiate J, Júlio E. Arbitrary bi-dimensional finite strain cohesive crack propagation. Comput Mech. 2009; 45(1):61–75.

[37] Bittencourt TN, Ingraffea AR, Wawrzynek PA, Sousa JL. Quasi-automatic simulation of crack propagation for 2D LEFM problems. Eng Fract Mech. 1996; 55(2):321–334.

[38] Colombo D, Giglio M. A methodology for automatic crack propagation modelling in planar and shell FE models. Eng Fract Mech. 2006; 73: 490–504.

[39] Shi GH, Goodman RE. Generalization of two-dimensional discontinuous deformation analysis for forward modeling. Int J Numer Anal Meth Geomech. 1989; 13:359–80.

[40] Gu J, Zhao ZY. Considerations of the discontinuous deformation analysis on wave propagation. Int J Numer Anal Meth Geomech. 2009; 33(12): 1449–1465.

[41] Ning YJ, Yang J, An XM, Ma GW. Modelling rock fracturing and blast induced rock mass failure via advanced discretization within the discontinuous deformation analysis framework. Comput Geotech. 2010; 38(1): 40–49.

[42] Ning YJ, An XM, Ma GW. Footwall slope stability analysis with the numerical manifold method. Int J Rock Mech Min. 2011; 48: 964–75.

[43] Shi GH. Manifold method of material analysis. In: Transactions of the 9th army conference on applied mathematics and computing. Minneapolis (USA). 1992; 57–76.

[44] Ma GW, An XM, Zhang HH, Li LX. Modeling complex crack problems with numerical manifold method. Int J Fract. 2009; 156(1): 21–35.

[45] Zhang HH, Li LX, An XM, Ma GW. Numerical analysis of 2-D crack propagation problems using the numerical manifold method. Eng Anal Bound Elem. 2010; 34(1):41–50.

[46] Cao RH, Cao P, Lin H, Ma GW, Fan X, Xiong XG. Mechanical behavior of an opening in a jointed rock-like specimen under uniaxial loading: Experimental studies and particle mechanics approach. Archives of civil and mechanical engineering. 2018; 18: 198–214.

[47] Lee H, Jeon S. An experimental and numerical study of fracture coalescence in pre-cracked specimens under uniaxial compression. Int. J. Solids Struct. 2011; 48 (6): 979–999.

[48] Yang SQ, Huang YH, Jing HW, Liu XR. Discrete element modeling on fracture coalescence behavior of red sandstone containing two unparallel fissures under uniaxial compression. Engineering Geology. 2014; 178: 28–48.

[49] Wong LNY, Zhang XP. Size Effects on Cracking Behavior of Flaw-Containing Specimens Under Compressive Loading. Rock Mechanics and Rock Engineering. 2014; 47(5): 1921–1930.

[50] Potyondy DO. Simulating stress corrosion with a bonded-particle model for rock. Int. J. Rock Mech. Min. Sci. 2007; 44 (5): 677–691.

[51] Cho N, Martin C, Sego D. A clumped particle model for rock. Int J Rock Mech Min Sci. 2007; 44(7): 997–1010.

[52] Wong LNY, Einstein HH. Crack coalescence in molded gypsum and Carrara marble: Part 1. Macroscopic observations and interpretation. Rock Mechanics and Rock Engineering. 2009; 42(3):475–511.

[53] Park CH, Bobet A. Crack initiation, propagation and coalescence from frictional flaws in uniaxial compression. Eng Fract Mech. 2010; 77(14):2727–2748.

[54] Xie H, Ju Y, Li L. Criteria for strength and structural failure of rocks based on energy dissipation and energy release principles. Chinese J Rock Mech Eng. 2005; 24(17):3003–10.

[55] Liang CY, Li X, Wang SH, Li SD, He JM, Ma CF. Experimental investigations on rate-dependent stress-strain characteristics and energy mechanism of rock under uniaixal compression. Chinese J Rock Mech Eng. 2012; 31 (9):1830–8.

[56] Itasca CG. Users’ Manual for Particle Flow Code in 2 Dimensions (PFC2D), Version. 2002

